# S-ketamine dampens effector T-cell function, thereby promoting tumor progression

**DOI:** 10.3389/fimmu.2026.1852040

**Published:** 2026-08-24

**Authors:** Dongge Niu, Lei Wang, Ruiyuan Wei, Lan Yao

**Affiliations:** Department of Anesthesiology, Peking University International Hospital, Beijing, China

**Keywords:** apoptosis, cancer pain, CAR-T cells, effector T cells, S-ketamine, TCR-T cells, tumor microenvironment

## Abstract

**Background:**

S-ketamine, a widely used clinical analgesic drug and also employed as a therapeutic regimen in cancer pain management, but whether S-ketamine influence on effector immune cells when conquering tumor cells remains unclear.

**Methods:**

In this study, we established effector T cells (chimeric antigen receptor T cells (CAR- T cells) and TCR-engineered T cells (TCR-T cells)) and treated with different concentrations of S-ketamine and investigated its impact on antitumor efficacy of effective T cell *in vitro* and *in vivo* assays.

**Results:**

Our findings demonstrated that S-ketamine treatment induced apoptosis in CAR- T and TCR-T cells, with higher concentrations leading to significant cell death. Furthermore, during coculture with target cells, increasing S-ketamine concentrations progressively dampened the early and late activation, impaired the tumor-killing capacity of both CAR-T and TCR-T cells, accompanied by reduced secretion of effector cytokines— particularly a striking downregulation of TNF-α production. Meanwhile, the expression of immune checkpoint receptors in T cells was upregulation under target cell stimulation and S- ketamine treatment. Consistent with in vitro findings, escalating S- ketamine concentrations dampened the tumor inhibition of TCR-T cells in a xenograft tumor model, and also diminished the tumor-infiltrating capability of TCR-T cells.

**Conclusions:**

our study reveals that S- ketamine suppresses the cytotoxic function and promotes exhaustive phenotype of effector T cells when conquering the target cells, and providing some new guides in clinical use of S-ketamine on cancer pain therapy

## 1 Introduction

Currently, the gradual increase in the incidence and mortality of tumors has become a significant burden on healthcare systems worldwide (1). In China, the incidence and mortality of tumors show a similar rising trend with the improvement in living standards and life expectancy (2, 3). The development and progression of tumors not only impose substantial health burdens on patients but also cause physical and psychological distress due to associated chronic pain—cancer pain (4, 5). Cancer pain is one of the most common and debilitating symptoms among cancer patients, severely affecting patients’ physical and mental well-being and may even lead to a loss of will. An effective regimen for cancer pain management is crucial for improving patients’ quality of life and survival time (5). Cancer pain therapies involve the use of different pharmacological interventions tailored to specific pain intensity levels. S-ketamine, as one of the options in cancer pain management, is widely utilized due to its remarkable analgesic effects. Previous studies have indicated that high concentrations of S-ketamine might inhibit tumor growth *in vitro* (6, 7). However, the tumor microenvironment involves dynamic interactions among immune cells, stromal cells, and tumor cells, all of which influence tumorigenesis and progression (8). The impact of S-ketamine on the tumor microenvironment remains poorly understood.

The tumor microenvironment (TME) constitutes a dynamic ecosystem encompassing immune cells, stromal cells, tumor cells, and other cellular subsets (9). Extensive research has established that immune cells orchestrate tumor evolution through a triphasic process—namely, elimination, equilibrium, and escape. The breakdown of the first two phases enables tumor cells to circumvent immune surveillance, thereby imposing a substantial health burden on patients (10). In this context, effector T cells, as key mediators of antitumor immunity, play a decisive role in restraining tumor growth (11). However, under chronic antigen stimulation in the tumor microenvironment, effector T cells gradually differentiate into exhausted T cells, characterized by upregulated expression of inhibitory receptors, altered metabolic and epigenetic profiles, and diminished cytotoxic capacity, ultimately resulting in tumor progression (12). In recent years, immune checkpoint blockade (ICB) therapy has been employed to reinvigorate T-cell function by antagonizing inhibitory receptors, thereby restoring the antitumor function of T cells. Furthermore, T-cell exhaustion under chronic stimulation in the tumor microenvironment has garnered considerable interest as a key determinant of therapeutic outcomes in cancer immunotherapy (13, 14). Therefore, it is imperative to investigate whether S-ketamine influences intratumoral T-cell function during cancer pain management.

In this study, we employed chimeric antigen receptor T cells (CAR-T cells) or TCR-engineered T cells (TCR-T cells) to model human effector T cells and examined the effects of S-ketamine at varying concentrations on effector T-cell function. Our results demonstrated that high concentrations of S-ketamine promoted T-cell apoptosis. Although low concentrations of S-ketamine caused minimal cytotoxicity to T cells, they significantly suppressed effector function during target cell coculture and upregulated the expression of immune checkpoint molecules. In tumor-bearing mice, escalating S-ketamine concentrations progressively attenuated the antitumor efficacy and infiltration capacity of effector T cells. The *in vitro* and *in vivo* findings suggested that the dose of S-ketamine should be carefully optimized in clinical applications to minimize its adverse effects on intratumoral effector T cells.

## 2 Materials and methods

### 2.1 Cell lines and culture

Lenti-X-293T cells for lentivirus packaging were purchased from Takara Biomedical Technology Co. Ltd. (Beijing, China). The Lenti-X 293T cell line is a subclone of the transformed human embryonic kidney cell line, HEK 293, which is highly transfectable and supports high levels of viral protein expression. The Lenti-X-293T cells were cultured in high glucose of Dulbecco's Modified Eagle Medium (DMEM) supplemented with 10% fetal bovine serum (FBS; HyClone, USA). A549 cells are a widely used human alveolar basal epithelial cell line. The A549 cells used in this research overexpressed HLA-A02:01, NYESO-1, and luciferase-GFP (A549^NYESO-1^) or overexpressed EGFR-VIII and luciferase-GFP (A549^EGFR-VIII^), and both were gifts from Xin Lin’s laboratory (Tsinghua University, China). Both A549^NYESO-1^ and A549^EGFRVIII^ cells were cultured in DMEM with 10% FBS and passaged every 48 h. All cells were cultured at 37°C in 5% CO_2_ and regularly tested for mycoplasma contamination using polymerase chain reaction (PCR; Forward, CGCCTGAGTAGTACGTTCGC, Reverse, GCGGTGTGTACAAGACCCGA) and consistently showed negative results.

### 2.2 TCR-T and CAR construction

The TCR-T receptor was constructed as described previously (15), using mutated murine TCR-α and TCR-β constant regions, with a variable region that targets the NYESO-1 peptide (TCR-T^NY-ESO-1^). CAR constructs comprised antigen-binding scFvs derived from clone 139 (anti-EGFRVIII)) (16), fused to a CD8 transmembrane domain and intracellular signaling domains from CD3ζ and 4-1BB (139-BBzCAR). For lentiviral delivery, TCR-T and CAR constructs were cloned into pHAGE vectors together with a red fluorescent protein (RFP) reporter.

### 2.3 Lentivirus production

A lentivirus vector (pHAGE, Addgene, USA) was used in our study, which contained an IRES-linked RFP reporter. The pHAGE vectors encoding 139-BBz CAR and NYESO-1 TCR contained a c-*myc* tag. For lentivirus packaging, a mixture of pHAGE vector, the packaging plasmid psPAX2 (Addgene), and the envelope plasmid pMD2.G (Addgene) was used at a 3:2:1 ratio and transfected into Lenti-X-293T cells using polyethyleneimine. The virus-containing culture medium supernatant was collected at 48- and 72-h posttransfection. The virus was mixed with PEG8000 (Sigma, USA) overnight at 4°C and centrifuged for 30 min. All viruses were stored at − 80°C for later use.

### 2.4 Preparation of CAR-T cells and TCR-T cells

Peripheral blood mononuclear cells (PBMCs) were separated from healthy donors. The collection and use of human PBMCs were approved by the Ethics Committee of Beijing Gaobo Boren Hospital (Approval Number: KY2023-002-005). Written informed consent was obtained from all healthy volunteers prior to blood collection. PBMCs were stimulated with 5 μg/mL anti-CD3 (OKT3, BioLegend, USA), 1 μg/mL anti-CD28 (BD Biosciences, USA), and 5 μg/mL fibronectin (Roche, Switzerland) for 24 h in RPMI-1640 medium supplemented with heat-inactivated 10% FBS and interleukin (IL)-2 (100 IU/mL). After 24 h of activation, T cells were infected with CAR or TCR lentivirus for 36 h, after which the medium was replaced with fresh medium. Four days later, the transduction efficiency was determined using FCM, and RFP positive T cells were sorted for *in vitro* assays.

### 2.5 CAR and TCR surface display ability assessment

Transduced T cells were collected and centrifuged for 5 min, and the supernatant was removed. The cells were then resuspended in PBSF (PBS containing 1% fetal bovine serum (FBS) and 0.5mM EDTA) and incubated with 1 μg/mL anti-c-*myc*-FITC antibody protein (BioLegend) on ice for 30 min. After washing twice with PBSF, the cells were subjected to flow cytometry analysis.

### 2.6 Cytotoxicity assay

The toxicity test of S-ketamine-treated tumor cells and cytotoxicity of T cells transduced with a CAR or TCR was determined by the Firefly Luciferase Reporter Gene Assay Kit (Vazyme, China). For the toxicity test of S-ketamine-treated tumor cells, 1 × 105 tumor cells were cocultured in flat-bottom 96-well plates and treated with different concentrations of S-ketamine for 24 h. For the effector/target ratio assay, the transduced T cells and tumor cells (A549^NY-ESO-1–1^ or A549^EGFR-VIII^) were cocultured in 1:1 effector/target ratios using flat-bottom 96-well plates with 1 × 105 target cells with or without different concentrations of S-ketamine for 24 h. After 24 h of treatment, the supernatant was collected for ELISA. The cells were lysed for 15 min, luciferase was released into the supernatant, and the firefly luciferase substrate was added before being monitored by the GloMax^®^-Multi + Detection System. The intensity of fluorescence is proportional to the viability of tumor cells. Percent cytotoxicity was determined as 100 × (experimental fluorescent intensity)/(negative control fluorescent intensity).

### 2.7 Enzyme-linked immunosorbent assay

After the transduced T cells and tumor cells were cocultured, the cytokine concentration in the culture medium was measured using the following ELISA kits: human IL-2 (eBioscience, USA), human interferon gamma (IFN-γ; eBioscience, USA), and human tumor necrosis factor alpha (TNF-α; eBioscience, USA). All samples were measured in triplicate according to the manufacturer’s protocol.

### 2.8 Flow cytometry

The following fluorophore-conjugated antibodies were used for cell surface molecule staining in PBSF: CD3-PE-cy7 (BioLegend), CD3-APC (BioLegend), CD25-APC (BioLegend), CD69-FITC (BioLegend), Programmed death receptor 1 (PD-1)-Percp/cy5.5 (BioLegend), Lymphocyte-activation gene 3 (LAG-3)-AF647 (BD Biosciences), Human Fc Block (BD Biosciences), and Fixable Viability Dye-eFluor506 (eBioscience, USA). Flow cytometry data were acquired by a BD Fortessa 4 laser and analyzed using FlowJo software (FlowJo, LLC, USA).

### 2.9 Cell morphology change and apoptosis

Transduced T-cell apoptosis was investigated by morphological observation or using the Annexin V-FITC/PI apoptosis detection kit (KeyGEN) combined with a flow cytometry assay. After transduced T cells were treated with different concentrations of S-ketamine for 24 h, the morphology of transduced T cell observed under a microscope, or the cell apoptosis was stained with Annexin V-FITC following standard protocols and immediately subjected to BD Fortessa (BD Biosciences). The results were analyzed using the FlowJo software (BD Biosciences).

### 2.10 T-cell checkpoint receptor expression detection

Transduced T-cells were pretreated with different concentrations of S-ketamine for 24 h, washed with DMEM, and the pretreated T cells were cocultured with 3 × 10^5^ target cells (E:T ratio = 1:3) in 96-well plates. After 24 h of coculture, the cells were collected and stained with surface markers following standard protocols. T-cell checkpoint receptor expression was assessed using a BD Fortessa 4 and analyzed using FlowJo software.

### 2.11 Animal assays

Male and female 6- to 8-week-old NOD/ShiLtJGpt-Prkdc^em26Cd52^IL2rg^em26Cd2^2/Gpt (NCG) mice were purchased from GemPharmatech Co. Ltd. (China). All mouse experiments were approved by the Institutional Animal Care and Use Committee (IACUC) of the Chinese Institute for Brain Research, Beijing (Approval Number: CIBR-IACUC-042). Animals were housed in a specific-pathogen-free (SPF) facility at Beijing Morningrise Hi-Tech Biotechnology Co. Ltd. (China), maintained under a 12-h light/dark cycle at 22 °C–24 °C with 40%–60% humidity, and provided with standard feed and water ad libitum. Environmental enrichment, such as nesting materials, was provided in all cages. For the subcutaneous tumor model, mice were inoculated with 5 × 10^4^ A549^NY-ESO-1–1^ tumor cells mixed with Matrigel (BD Biosciences) via subcutaneous injection in a 200-μL volume on day 0. After 6 days, 2.5 × 10^6^ transduced T cells or mock-transduced T cells were infused intravenously. Subsequently, varying concentrations of S-ketamine were injected into the mice. No anesthesia or analgesia was administered during the routine subcutaneous or intravenous injections, as these procedures caused only transient and mild distress. Tumor progression was measured by calipers in three dimensions (*V* = *L* × *W* × *H*).

To minimize animal suffering, humane endpoints were strictly enforced. Mice were euthanized early if the maximum dimension of the subcutaneous tumor reached 2.0 cm or if they exhibited severe signs of distress (e.g., weight loss exceeding 20%, lethargy, or inability to access food/water). The planned experimental endpoint was determined by a tumor volume *V* > 1,000 mm^3^. At the conclusion of the experiment or upon reaching humane endpoints, all surviving mice were humanely euthanized by cervical dislocation following deep CO_2_ anesthesia to ensure minimal suffering, and different organs were isolated for subsequent analyses. No randomization or blinding methods were used.

### 2.12 Histological staining

For histopathological analyses, tumor tissue was fixed in 4% paraformaldehyde solution, processed according to standard procedures, embedded in paraffin, and sectioned. Five-micrometer-thick sections were incubated with a primary antibody specific to human CD3 (Abcam, UK) and then with a peroxidase-labeled secondary antibody, and images were acquired using the 3DHistech Pannoramic SCAN. Subsequently, CaseViewer software was applied to evaluate the quantity of T cells in tumor tissue.

### 2.13 Statistical analysis

All statistical analyses were performed using SPSS software. No statistical methods were used to predetermine the sample size. Statistical comparisons between multiple groups were determined by one-way ANOVA. *p*-value < 0.05 was considered to be statistically significant.

## 3 Results

### 3.1 TCR-T and CAR-T cells exhibit potent tumor inhibition

S-ketamine is a widely used clinical analgesic drug in cancer pain management, demonstrating outstanding efficacy in analgesia. Although some *in vitro* studies have shown that high-concentration S-ketamine treatment can induce apoptosis in tumor and effector immune cells (17–19), it is noteworthy that current research has not yet explored the effects of S-ketamine on the intratumoral immune microenvironment, particularly its impact on T-cell effector functions. This knowledge gap warrants systematic investigation to evaluate both the therapeutic benefits and potential immunomodulatory implications of S-ketamine in cancer pain applications. Therefore, the purpose of this study is to analyze the effects of different concentrations of S-ketamine on T-cell effector function under tumor cell stimulation.

**Figure 1:**
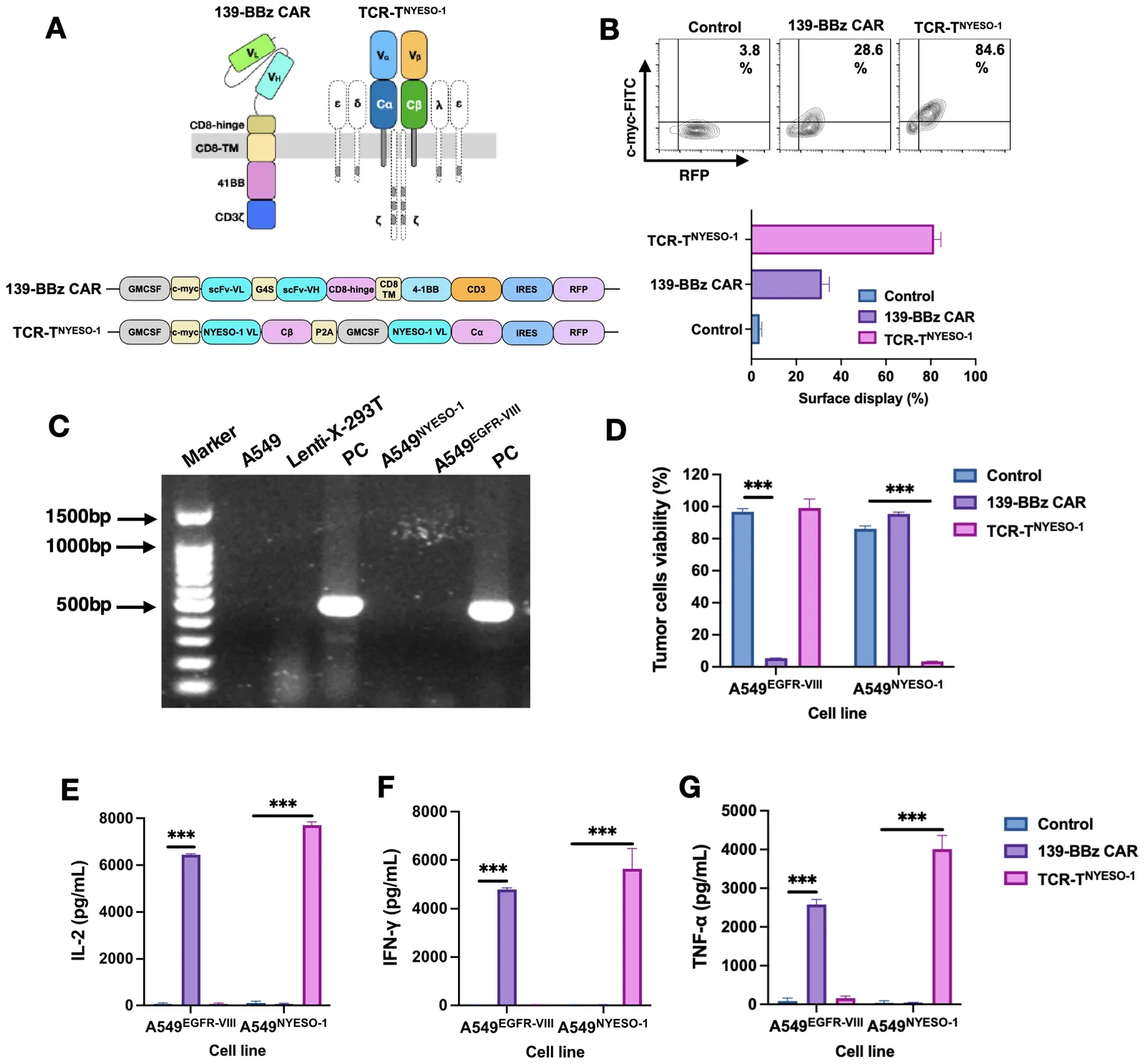
Functional validation of EGFR-VIII-targeted 139-BBzCAR-T cells and NYESO-1-targeted TCR-T cells. **(A)** Schematic diagrams of the EGFR-VIII-targeted CAR and NYESO-1-targeted TCR. The upper panels illustrate the structural designs, while the lower panels show plasmid schematics for each construct. **(B)** Surface expression levels of the 139-BBzCAR and TCR-T^NYESO-1^ constructs in human primary T cells after lentiviral infection. Top, flow cytometry analysis; bottom, bar graphs quantifying the surface expression level of each construct in T cells from different donors. **(C)** Mycoplasma testing for each cell line using PCR, where PC represents the positive control. **(D)** Cytotoxic activity of 139-BBzCAR-T cells against A549^EGFR-VIII^ target cells and TCR-T^NYESO-1^ cells against A549^NYESO-1^ target cells. **(E–G)** Cytokine secretion profiles after coculture of 139-BBzCAR-T cells with A549^EGFR-VIII^ target cells or TCR-T^NYESO-1^ cells with A549^NYESO-1^ target cells: **(E)** IL-2 secretion, **(F)** IFN-γ secretion, and **(G)** TNF-α secretion. Statistical significance was determined by one-way ANOVA followed by Tukey’s multiple comparisons test. *p < 0.05, **p < 0.01, ***p < 0.001 vs. control group.

CAR-T and TCR-T cells were ideal effector T cells to target tumor cells, so we constructed two plasmids: one encoding a 139-BBzCAR targeting epidermal growth factor receptor variant III (EGFR-VIII), and the other encoding a TCR-T^NY-ESO-1^ targeting New York esophageal squamous carcinoma-1 (NY-ESO-1) peptide, which is presented by HLA02:01 (**Figure 1A**). After packaging the lentiviruses for 139-BBzCAR and TCR-T^NYESO-1^, we used the lentiviruses to infect T cells from different healthy donors. Subsequently, we performed flow cytometry to detect the surface display of 139-BBz CAR and TCR^NYESO-1^ on T cells and found that 39-BBz CAR and TCR^NY-ESO-1^ could be efficiently expressed on the T-cell membrane (**Figure 1B**). Next, we sorted the positive T cells and cocultured 139-BBzCAR-T cells and TCR-T^NYESO-1^ cells with A549^EGFR-VIII^ target cells and A549^NYESO-1^ target cells, respectively. Before we carried out the functional assays, we performed mycoplasma testing by PCR; the results indicated that each target cell line was free of mycoplasma contamination (**Figure 1C**). After that, we cocultured 139-BBzCAR-T cells and TCR-T^NYESO-1^ cells with the target cells to detect T-cell cytotoxicity. The cytotoxicity results demonstrated that 139-BBzCAR-T cells and TCR-T^NYESO-1^ cells could effectively kill their respective target cells (**Figure 1D**) and secrete effector cytokines (**Figures 1E–G**). From these results, we concluded that the prepared CAR-T cells and TCR-T cells were capable of effectively killing target cells, providing a solid foundation for subsequent research on how S-ketamine affects the function of effector T cells.

### 3.2 S-ketamine induces CAR-T- and TCR-T-cell apoptosis

**Figure 2:**
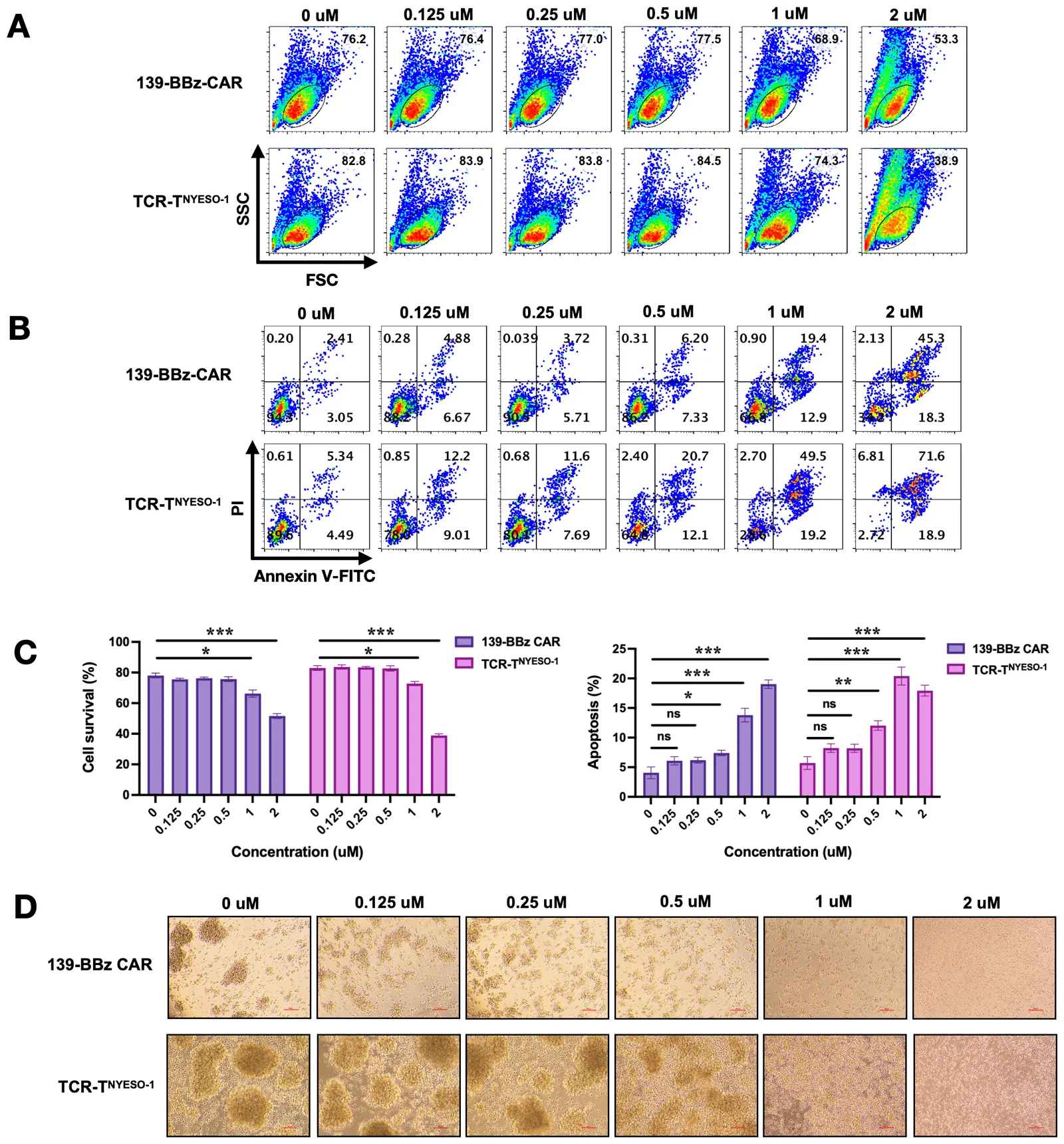
Effects of different concentrations of S-ketamine on CAR-T and TCR-T cells. **(A)** Flow cytometry analysis of cell viability for CAR-T and TCR-T cells after 24 h of treatment with different concentrations of S-ketamine. **(B)** Flow cytometry analysis was employed to evaluate apoptosis in CAR-T and TCR-T cells after 24 h of treatment with varying concentrations of S-ketamine. **(C)** Bar graph analysis of cell viability and apoptosis: left, the viability of CAR-T and TCR-T cells after S-ketamine treatment; right, the apoptosis levels of these cells after treatment. **(D)** Microscopic images of CAR-T and TCR-T cells after 24 h of exposure to different concentrations of S-ketamine. Statistical significance was determined by one-way ANOVA followed by Tukey’s multiple comparisons test. *p < 0.05, **p < 0.01, ***p < 0.001 vs. control group.

Based on the aforementioned results, we treated the 139-BBzCAR T cells and TCR-T^NYESO-1^ cells with different concentrations of S-ketamine and monitored the effects of S-ketamine on the T cells. After treating CAR-T cells and TCR-T cells with different concentrations of S-ketamine for 24 h, we analyzed the viability of CAR-T cells and TCR-T cells after S-ketamine treatment by flow cytometry. Flow cytometry results revealed that S-ketamine concentrations ranging from 1 to 2 μM significantly affected the survival of T cells, while those ranging from 0.125 to 0.5 μM had no significant impact on T-cell viability (**Figures 2A, C** [left]). Furthermore, we further validated the effects of S-ketamine on T cells by detecting apoptosis. Apoptosis results demonstrated that 1 to 2 μM S-ketamine significantly induced T-cell apoptosis and led to cell death, whereas 0.125 to 0.25 μM S-ketamine did not significantly trigger T-cell apoptosis (**Figures 2B, C** [right]). Meanwhile, treatment with 0.5 μM S-ketamine did not induce significant cell death in CAR-T or TCR-T cells but slightly triggered apoptosis. Additionally, microscopic observations further confirmed that 0.125 to 0.25 μM S-ketamine had no significant impact on T cells during culture, as the T cells continued to grow in clusters and maintained a healthy growth state. However, at concentrations of 1 to 2 μM S-ketamine, both CAR-T and TCR-T cells failed to form clusters and exhibited significant apoptotic morphology (**Figure 2D**). Therefore, we confirmed that increasing the concentration of S-ketamine could induce T-cell apoptosis and even cell death, but treatment with low concentrations of S-ketamine did not significantly affect the physiological state of T cells without stimulation.

### 3.3 S-ketamine inhibits CAR-T and TCR-T cells’ effector function and promotes exhaustion with increasing concentrations

**Figure 3:**
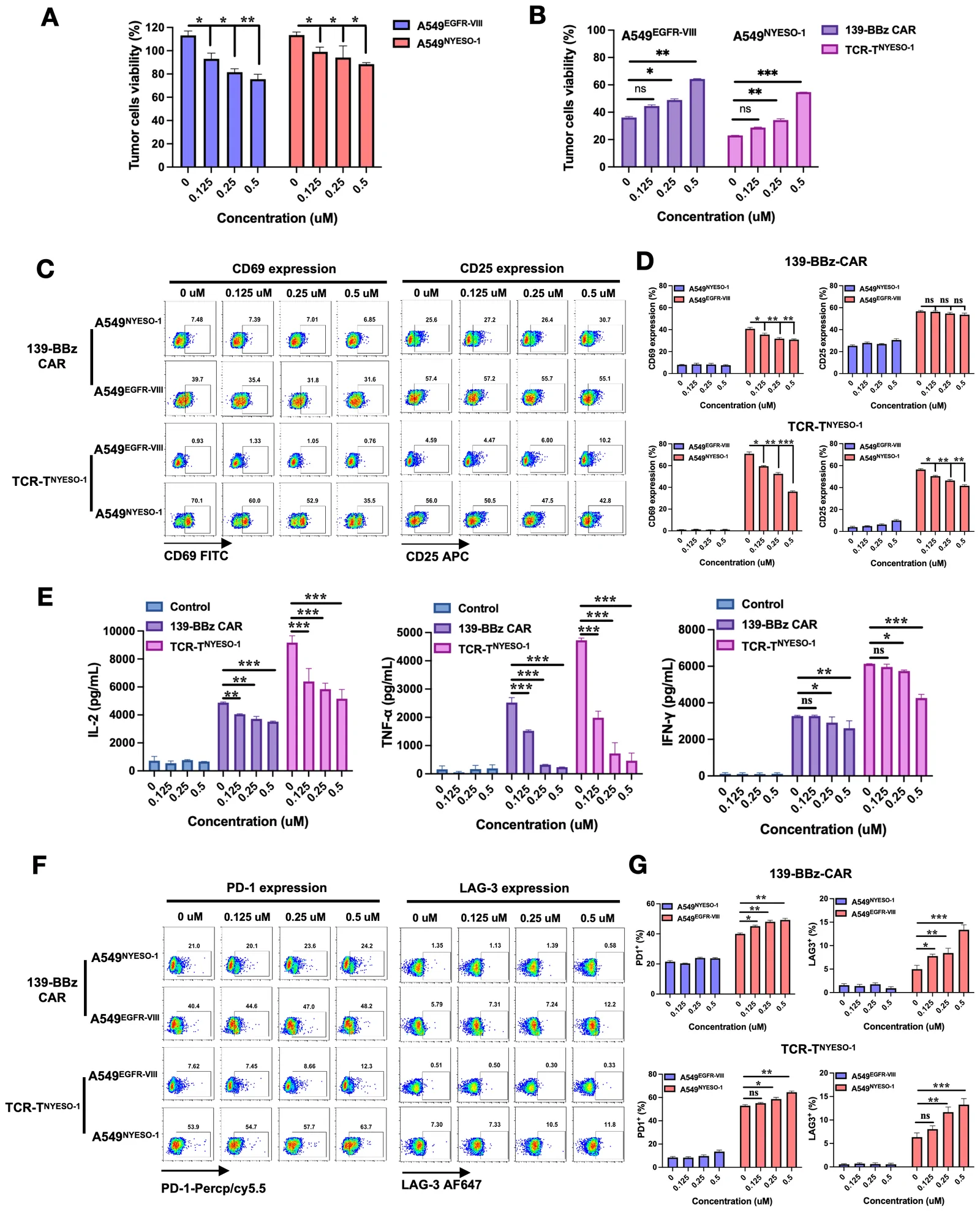
The functional impact of S-ketamine at different concentrations on CAR-T cells and TCR-T cells. **(A)** Target cell survival under S-ketamine treatment. **(B)** 139-BBzCAR-T and TCR-T^NYESO-1^ cells mediated the killing of target cells after S-ketamine pretreatment for 24 h. **(C)** After treatment with different concentrations of S-ketamine for 24 h, flow cytometric analysis was performed to examine the activation status of CAR-T cells and TCR-T cells in S-ketamine-free culture medium. The expression levels of the early activation marker (CD69) and the late activation marker (CD25) were detected. **(D)** Bar graph analysis of CD69 and CD25 expression. **(E)** After treating T cells with different concentrations of S-ketamine for 24 h, the secretion of various cytokines was examined after 18 h of coculture of CAR-T cells and TCR-T cells with target cells in S-ketamine-free culture medium. **(F)** After pretreating T cells with S-ketamine for 24 h, the expression levels of the exhaustion markers PD-1 and LAG-3 were analyzed using flow cytometry to assess T-cell exhaustion. **(G)** The expression levels of PD-1 and LAG-3 were analyzed using bar graphs. The top bar graph shows the expression of PD-1 and LAG-3 in CAR-T cells, while the bottom bar graph shows the expression of PD-1 and LAG-3 in TCR-T cells.

Previous works have indicated that human hepatoma HepG2 cells and human pancreatic adenocarcinoma Panc-1 cells treated with high concentrations of S-ketamine could undergo apoptosis or necrosis (17, 18). We treated target cells with different concentrations of S-ketamine and confirmed that S-ketamine could lead to target cell death (**Figure 3A**). To avoid the influence of S-ketamine on target cells, we pretreated CAR-T and TCR-T cells with different concentrations of S-ketamine for 24 h and detected the functional changes of CAR-T and TCR-T cells when cocultured with target cells. After coculturing with target cells for 24 h, cytotoxicity results revealed that S-ketamine could dampen T-cell killing ability with increasing concentrations (**Figure 3B**). Based on the cytotoxicity results, we wondered whether S-ketamine would impact effector T-cell activation and thereby dampen T-cell function. Therefore, after S-ketamine pretreated effector T cells for 24 h, T cells were stimulated by target cells at different time points. We next detected cluster of differentiation (CD)69 and CD25 expression, which were considered early and late markers of lymphocyte activation, respectively (20). The flow cytometry results indicated that the activation levels of both CAR-T cells and TCR-T cells were significantly impaired by S-ketamine, regardless of whether early or late activation markers were examined (**Figures 3C, D**). Additionally, after S-ketamine pretreatment, the secretion of functional cytokines from CAR-T or TCR-T cells was significantly reduced under target cell stimulation, especially for TNF-α (**Figure 3E**). High expression of T-cell exhaustion receptors is a critical factor that severely impairs effector T-cell function and contributes to tumor cells escaping T-cell surveillance. Therefore, we also examined the expression of exhaustion markers PD-1 and LAG-3 on CAR-T cells and TCR-T cells after coculture with target cells following S-ketamine pretreatment. Flow cytometry analysis showed that S-ketamine significantly increased the expression of PD-1 and LAG-3 on both CAR-T and TCR-T cells with increasing concentrations. Notably, PD-1 and LAG-3 expression in CAR-T cells increased with each dose, but TCR-T cells showed no significant increase at 0.125 μM (**Figures 3F, G**). These findings suggested that a low concentration of S-ketamine still impaired T-cell effector function, implying that during analgesia in cancer patients, S-ketamine may affect the effector function and accelerate T-cell exhaustion within tumors, potentially promoting tumor growth.

### 3.4 Low concentrations of S-ketamine affected the cytotoxicity of TCR-T cells *in vivo*

**Figure 4:**
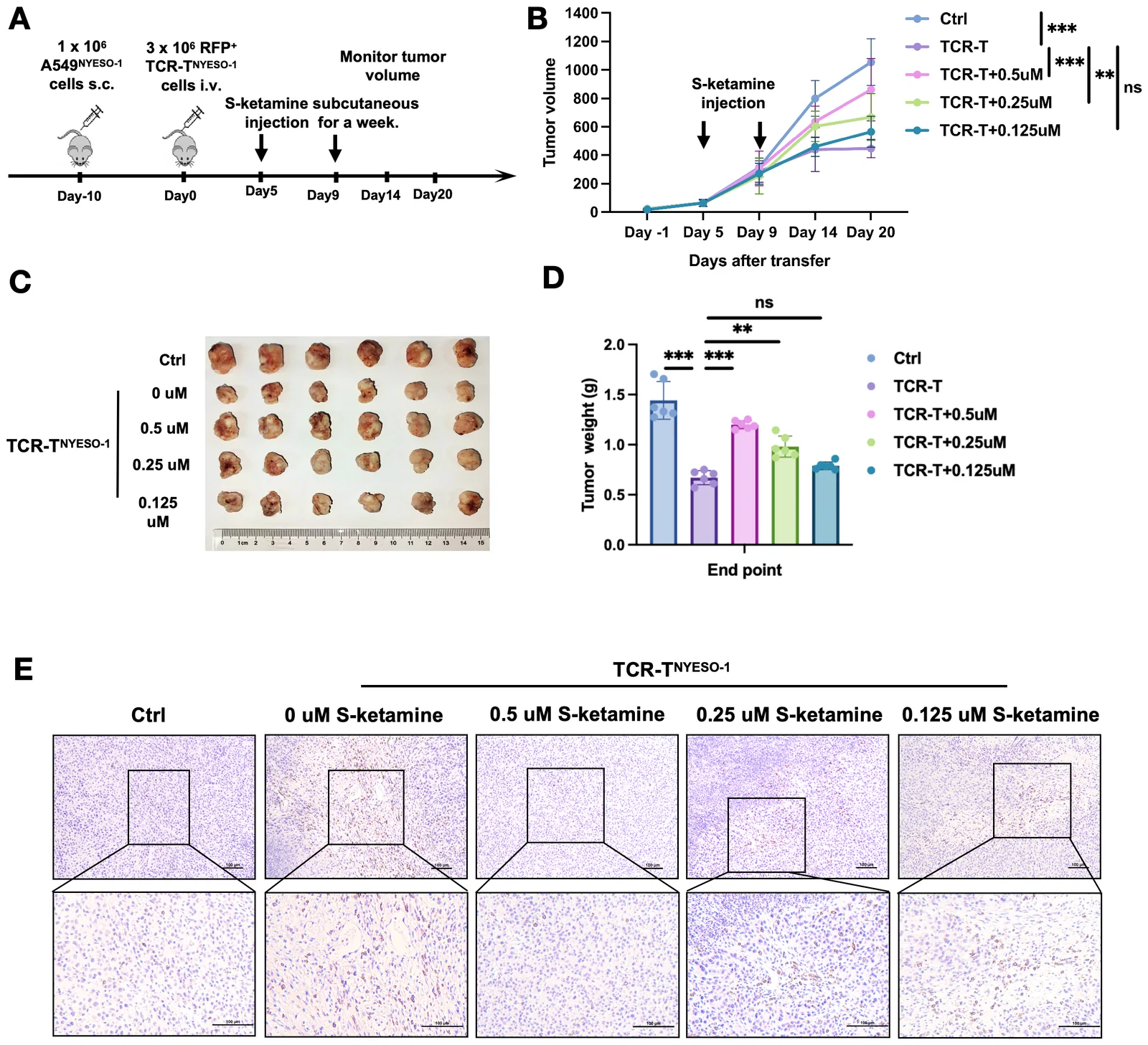
S-ketamine inhibits the antitumor functional ability of TCR-T cells *in vivo*. **(A)** Schematic diagram illustrating the monitoring of the antitumor functional ability of TCR-T^NYESO-1^ cells treated with S-ketamine in the A549^NYESO-1^ xenograft tumor model. **(B)** Tumor volume (*V* = *L* × *W* × *H*) measured at different time points. **(C)** Images of tumors collected on day 20. **(D)** Tumor weight for each experimental group on day 20. **(E)** Immunohistochemical staining of CD3 T cells in the tumor tissue (scale bar = 100 μm). Statistical significance was determined by one-way ANOVA followed by Tukey’s multiple comparisons test. *p < 0.05, **p < 0.01, ***p < 0.001 vs. control group.

To investigate the effect of S-ketamine on the antitumor activity of effector T cells, and given the limited infiltration capacity of CAR-T cells in solid tumors and that TCR-T cells are major immune cells used to target tumor cells in patients (21, 22), we subcutaneously inoculated immunodeficient mice (NCG mice) with A549^NYESO-1^ antigen to establish a lung cancer-bearing mouse model and infused TCR-T^NYESO-1^ cells targeting the NYESO-1 antigen to detect the effect of S-ketamine on T-cell function. After TCR-T^NYESO-1^ cell inoculation for a week, we injected a total volume of 50 μL of S-ketamine at different concentrations into multiple sites around the tumor (**Figure 4A**). We then detected tumor volume at different time points to monitor the effects of S-ketamine on the antitumor function of TCR-T^NYESO-1^ cells. Under different concentrations of S-ketamine treatment, the antitumor activity of TCR-T^NYESO-1^ cells gradually decreased as the concentration of S-ketamine increased. However, it is noteworthy that at a low concentration of S-ketamine (0.125 μM), the antitumor activity of TCR-T^NYESO-1^ cells exhibited no significant difference compared to the untreated group (**Figure 4B**). At the experimental endpoint, we sacrificed the mice to obtain tumor tissue and measured the tumor volume and weight. Similarly, we found no significant difference in tumor volume between the low-concentration S-ketamine-treated group and the untreated group. However, the tumor volume in the high-concentration (0.5 μM) S-ketamine-treated group was significantly larger (approximately 50% larger) than those in the untreated and low-concentration (0.125 μM) treated groups (**Figures 4C, D**). Subsequently, we performed immunohistochemical experiments to detect the abundance of T cells within the tumors in different groups. The results also indicated that with increasing S-ketamine concentrations, the number of TCR-T^NYESO-1^ cells within the tumors gradually decreased, with significantly fewer T cells in the 0.25- and 0.5-μM treatment groups compared to the low-concentration S-ketamine-treated group (**Figure 4E**). These findings suggested that the use of S-ketamine in cancer patients with cancer pain may impair the function of T cells within the tumors, thereby promoting tumor progression.

## 4 Discussion

In clinical practice, nonsteroidal anti-inflammatory drugs (NSAIDs) are the first-line treatment for mild cancer pain. Beyond analgesia, NSAIDs may enhance chemotherapy sensitivity and suppress tumor cell proliferation, offering additional therapeutic benefits (23–25). Recently, NMDA receptor antagonists like S-ketamine have also been utilized for mild cancer pain. Previous studies suggest that high-dose S-ketamine can induce tumor cell apoptosis or necrosis (17, 18), potentially inhibiting tumor growth. Some research indicates that S-ketamine may dampen effector immune cell function, indirectly promoting tumor progression (26, 27). These findings suggest that the use of S-ketamine to treat cancer pain may influence tumor development. In this study, we demonstrate that S-ketamine administered to effector T cells (CAR-T or TCR-T cells) induces dose-dependent apoptosis (**Figure 2**). Under low-concentration treatment, S-ketamine progressively suppresses T-cell activation, cytotoxic capacity, and secretion of effector cytokines (e.g., TNF-α, IFN-γ). Additionally, S-ketamine upregulates exhaustion markers (e.g., PD-1, TIM-3) on effector T cells, accelerating dysfunction (**Figure 3**). In tumor-bearing mice, low-dose S-ketamine minimally affected TCR-T-cell cytotoxicity, but higher doses significantly inhibited antitumor efficacy and reduced tumor infiltration (**Figure 4**).

Effector T cells play a critical role in regulating tumor development and progression. Most studies investigating the effects of drugs on T-cell effector function rely on murine-derived OT-1 cells; however, the conclusions from these studies may not be directly translatable to clinical applications (28, 29). With the development of genetic engineering technology, CAR-T and TCR-T cells have been used to evaluate the toxicity of chemical compounds or drugs on effector T cells during antitumor responses (30, 31). Here, we successfully generated functional CAR-T and TCR-T cells as effector T-cell models (**Figure 1**).

Previous studies reported that S-ketamine could induce apoptosis or necrosis after treatment of effector immune cells. Consistent with these findings, we observed significant T-cell apoptosis at S-ketamine concentrations greater than 1 μM. However, S-ketamine doses from 0.125 to 0.5 μM did not significantly induce apoptosis (**Figure 2**), suggesting a dose-dependent proapoptotic effect. Chronic antigen exposure during antitumor responses drives T-cell dysfunction or exhaustion, impairing immune surveillance (12). Prior work suggests that S-ketamine suppresses effector T cells, potentially by inhibiting TNF-α secretion (32), thereby compromising antitumor immunity and promoting tumor progression. In our work, S-ketamine treatment during T-cell coculture with target cells also inhibited T-cell activation, cytotoxic function, and effector cytokine secretion. Notably, TNF-α production declined in a dose-dependent manner (**Figure 3**), suggesting that S-ketamine might act on signaling pathways to mediate TNF-α expression and influence T-cell effector function. Moreover, the most important finding of this study is that S-ketamine induced dose-dependent T-cell exhaustion; higher S-ketamine concentrations (but not 0.125 μM) upregulated exhaustion markers (PD-1, LAG-3). Furthermore, in tumor-bearing mice, increasing S-ketamine doses progressively reduced TCR-T-cell-mediated tumor killing and diminished tumor infiltration capacity, but low-dose S-ketamine (0.125 μM) treatment did not dampen the infiltration capacity and antitumor efficacy of TCR-T cells.

In conclusion, these results suggest that S-ketamine has a dose-dependent inhibitory effect on the functions of effector T cells. It may promote T-cell exhaustion, thereby reducing the cytotoxicity and tumor-infiltrating ability of T cells and promoting further tumor development. However, the inhibitory effect of S-ketamine may be attenuated as its concentration gradually decreases. Importantly, this study provides a scientific rationale for the rational use of S-ketamine in the clinical treatment of patients with cancer pain, enabling patients to achieve effective cancer pain relief without affecting the effectiveness of tumor treatment.

## Data Availability

The raw data supporting the conclusions of this article will be made available by the authors, without undue reservation.
